# Effect of Physical Activity on Drug Craving of Women With Substance Use Disorder in Compulsory Isolation: Mediating Effect of Internal Inhibition

**DOI:** 10.3389/fpsyg.2019.01928

**Published:** 2019-09-03

**Authors:** Kun Wang, Jiong Luo, Tingran Zhang, Yiyi Ouyang, Chenglin Zhou, Yingzhi Lu

**Affiliations:** ^1^Research Centre for Exercise Detoxification, College of Physical Education, Southwest University, Chongqing, China; ^2^School of Kinesiology, Shanghai University of Sport, Shanghai, China

**Keywords:** physical activity, substance use disorder, drug craving, internal inhibition, mediating effect

## Abstract

**Background:**

Women with substance use disorder has attracted widespread attention as a prominent social issue. According to reports, physical exercise can improve the internal inhibition, effectively reduce the substance user’s drug graving, and improve withdrawal symptoms, however, the specific mechanism of internal inhibition should be further considered. This study was designed to determine the critical role of internal inhibition in the path of physical exertion affecting the drug cravings of women drug users.

**Methods:**

By means of Physical Activity Rating Scale (PARS-3), internal Inhibition Scale and Drug Craving Scale, this study investigated the individuals with substance use disorder under rehabilitation in the women compulsory isolation rehabilitation center in Chongqing, China.

**Results:**

(1) Women with traditional drug users had the strongest internal inhibition and new drug use disorder had the highest drug craving. The longer the duration of drug abuse, the lower the internal inhibition and the higher the drug craving. Women with moderate-intensity activity had the strongest internal inhibition and the lowest drug craving. (2) The physical activity intensity was negatively correlated with drug craving, positively correlated with intrinsic inhibition, and negatively correlated with drug craving. (3) Internal inhibition played a partial mediating effect between physical activity intensity and drug craving.

**Conclusion:**

Physical activity has a positive effect on inhibiting drug craving among drug addicts, while moderate-intensity activity seems to be more conducive to enhancing the internal inhibition of addicts, to improving their resistance to drugs, and thus more conducive to reducing drug craving.

## Introduction

According to the *Word Drug Report 2018*, about 275 million individuals used drugs at least once in 2016 all over the world, which posing a huge threat to safety and health to humankind. It is well known that when an individual continues to use a certain drug and forms the dependence, it is easy to produce a drug craving and a related drug impulse behavior. Drug craving is the subjective craving of drug addicts or users for drug effects, and their inner expectations of pleasant experience, relieving withdrawal symptoms and negative emotions brought by drugs, and subconsciously paying too much attention to drug-related cues (Washton, 1986; Field et al., 2009). When an individual is craving for drugs, there will be a rift between his real self and his ought self, which will easily lead to negative emotions related to anxiety and depression (De los Cobos et al., 2011). The higher the degree of drug craving, the higher the level of anxiety and depression (Higgins, 1987). On the basis of the self-medication hypothesis, young individuals with high social anxiety may use marijuana to alleviate emotional distress and respond to unpleasant social conditions (Khantzian, 1997), and individuals with higher drug dependence are more likely to be paranoid, and then exacerbate psychotic-like symptoms (Musetti et al., 2016). At the same time, individuals with substance use disorder may actually use substance or excessive behavior as an external regulator and self-medication to treat their emotional disorders and painful mental states, and individuals with alcohol use disorders often suffers more traumatic events in childhood (Craparo et al., 2014; Caretti et al., 2018). Based on this, Schimmenti and Bifulco (2015) pointed out in the report that *the Childhood Experience of Care and Abuse* (CECA) has good psychometric characteristics and was considered to be the gold standard for assessing trauma and lack of care experienced during childhood, and these also achieved initial results in predicting depression. It can be seen that the psychological mechanism of drug addiction and addictive behavior is very extensive and has always been an important topic in academic circles.

In recent years, the role of physical activity in reducing drug craving has attracted wide attention. Among tobacco abusers, Roberts et al. (2012) found that aerobic activity can significantly reduce tobacco abusers’ craving for tobacco, while women tobacco abusers seem to be more likely to abandon tobacco than male tobacco abusers. Linke et al. (2013) pointed out that aerobic activity can effectively prevent or alleviate the negative emotional state when quitting smoking, and reduce their craving for tobacco. Physical activity can reduce the craving for smoking and improve the adverse symptoms after withdrawal. In a study of drug abstainers, such as cannabis and opioids, it was found that after 6 months, three times a week and 2 h of aerobic activity intervention, drug abstainers’ craving for drugs was significantly reduced (Roessler, 2010). Similarly, Buchowski et al. (2011) also found that aerobic exercise can effectively reduce the craving for marijuana by addicts. A review study has shown that the proper physical exercise can effectively inhibit the psychological cravings of the addicts and the corresponding relapse behavior through the regulation of neurotransmitters and hormones (Zhao et al., 2018). Strickland et al. (2016) pointed out that participation in sports can enhance the ability of dopaminergic signaling, especially in the “reward” approach, which can effectively reduce the excessive use of drugs by addicts. Some scholars hypothesized that physical exercise can reduce smoking dependence, and validate the hypothesis through the path model. It have found that among the college students who were addicted to smoking, the greater the amount of physical exercise, the lower the smoking dependence Chang et al. (2014). It can be seen that physical activity does play a positive role in reducing drug craving, but the specific path between these two is still unknown.

Internal inhibition is an important component of executive function of the brain. It intends to cancel a dominant response or stop an inappropriate ability unrelated to action. It is closely related to the prevention and treatment of smoking addiction, hyperactivity disorder, drug abuse and schizophrenia clinically (Schachar et al., 2000; Friedman and Miyake, 2003; Tomporowski et al., 2008). A large number of studies have found that: two consecutive weeks of self-control training can significantly prolong the duration of smoking cessation of the abstainer (Muraven et al., 1999). Individuals with low self-control level are more likely to have drug craving and abuse behavior, while those with high self-control level show less substance abuse behavior (Tarantino et al., 2015). Self-control ability of male drug addicts is one of the important influencing factors of relapse behavior. It is negatively correlated with relapse tendency (Gong et al., 2013). However, this result has not been verified in women with substance use disorder so far. The influence of physical activity on individual inhibition ability is also supported by more studies (Huang et al., 2014). Normal people who participated in aerobic activity performed better in the Flanker tasks related to inhibition, and the fronto-parietal network was more optimized in the execution of the task. Low-intensity of acute aerobic activity can promote the performance of the subjects when executing Stroop inhibits task, and effectively improve the activation level of dorsolateral prefrontal cortex and frontal polar brain area (Byun et al., 2014). However, acute aerobic exercise at moderate-intensity is better to promote human inhibition. Empirical studies have shown that both acute exercise and aerobic exercise can improve the inhibitory function of methamphetamine addicts (Buchowski et al., 2011). Although previous studies on the effects of physical exercise on the cravings and physical fitness of women with substance use disorder (see Table 1), whether physical exercise has the same effect on the inhibition ability of women drug users, and the relevant mechanism of action still needs further exploration. As known, acute aerobic activity at moderate-intensity can better promote human inhibition ability, and the response speed of inhibition task after moderate-intensity aerobic activity is significantly better than that of no activity and other activity intensity. Other studies have found that there is an inverted U-shaped relationship between acute aerobic activity and inhibition (Wang and Zhou, 2014).

**TABLE 1:** Research review on physical exercise on the drug craving and physical fitness in women with substance use disorder.

**Authors (Date)**	**Subjects**	**Protocol**	**Frequency**	**Outcome indicators**
Dolezal et al., 2013	Methamphetamine dependents (total of 39 of men and women aged 18–55)	Endurance and resistance exercise (treadmill, smith machine or dumbbell)	3 days/weeks, 1 h/times, total of 5 weeks	Significantly increased Vo_2max_, leg, and waist strength and endurance, significantly decreased fat
Rawson et al., 2015	Methamphetamine addicts (total of 135 of men and women aged 18–45)	Aerobic exercise and resistance training (aerobic for 30 min, resistance for 15 min)	3 days/weeks, 55 min/day, total of 8 weeks	Significant improvement in aerobic performance, heart rate variability, significantly decreased scores of depression and anxiety, decreased relapse rate
Geng et al., 2016	Synthetic drug force (total of 60 of women)	Taichi rehabilitation exercise (Wild horses branching, rewinding, kneeling, stalking, golden chicken independence and single whip)	5 days/weeks, 45 min/days, total of 3 months	Significant improvement in systolic pressure, balance ability, somatization disorder, depression, and anxiety
Wang et al., 2017	Methamphetamine dependents (men: 44, women: 6)	Aerobic exercise intervention (cycle ergometer, jogging, rope skipping) (warm-up: 5 min, aerobic exercise: 30 min, cool down: 5 min)	3 days/weeks, 30–40 min/days, total of 12 weeks	Effective improvement in physical fitness, craving degree and emotional disorder?
Caretti et al. (2018)	Clinical group (total 515 of women 111, men 398); Control group (total of 183, women 98, men 84)	the Severity Index (SI); the Seven Domains Addiction Scale (7DAS)	—	The better psychological assessment characteristics of The Addictive Behavior Questionnaire (ABQ)
Zhang et al., 2018	Amphetamine-type stimulant dependents (men: 76, women: 79)	Desire for Speed Questionnaire, DSQ; Sports Activity Rating Scale, PARS-3, etc.	—	Improving the negative emotions, mental state, and physical health, and then alleviate the psychological cravings of dependents
Zhu Dong et al., 2018	Amphetamine-type stimulant dependents (women: 80)	Taichi boxing (warm-up: 10 min, Taichi for 40 min, cool-down: 10 min)	5 days/weeks in first 3 months; 3 days/weeks in second 3 months	Increased sleep efficiency, significantly reduced pulse rate, significantly improved body fat content, significantly reduced withdrawal rate
Nygard et al., 2018	Methamphetamine addicts (men: 17, women: 6)	Resistance training (4 sets of half-squat exercises)	3 days/weeks, total of 12 weeks	Obviously upgrade in physical fitness
Liang et al., 2019	New drugs (Ice, ecstasy, K powder) abusers (women: 73)	Yoga and moderate-intensity aerobics (warm-up: 10 min, aerobics: 20 min, Yoga: 10 min)	3 days/weeks, ∼40 min/days, total of 3 months	Effective alleviated the negative emotion of depression and anxiety of subjects
Huang et al., 2019	Personnel in drug rehabilitation center (men: 7947, women : 790)	Physique measures (cardiopulmonary fitness, muscular strength fitness, and neural fitness, etc.)	—	Physical benefits not optimistic, and significant regional differences

**FIGURE 1:**
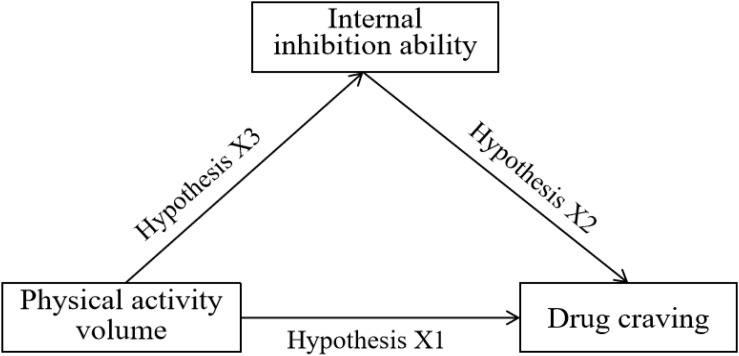
Hypothesis model diagram of mediating effect of internal inhibition ability on physical activity and drug craving.

In conclusion, physical activity plays a unique role in reducing drug users’ craving for drugs, improving depression and anxiety, and increasing drug withdrawal rate. Proper aerobic activity can promote the recovery of the impaired cognitive control ability of drug abusers, enhance the inhibition ability of drug craving, and then achieve the curative effect of rehabilitation. It can be seen that internal inhibition plays a key role between physical activity and drug addicts’ craving for drugs. Although most studies have confirmed that physical activity is closely related to internal inhibition, with the change of physical activity intensity, whether internal inhibition will change accordingly has been confirmed by few studies at present. Similarly, few studies have explored physical activity, internal inhibition and drug craving in the path model, especially for women with substance use disorder. Therefore, in view of the limitations of previous studies, this study proposes the following assumptions: (X1) Physical activity was negatively correlated with drug craving. (X2) Internal inhibition was negatively correlated with drug craving. (X3) Physical activity is positively correlated with internal inhibition. (X4) Internal inhibition mediates the physical activity and drug craving of women with substance use disorder (see Figure 1).

## Subjects and Methods

### Subjects

All the women with substance use disorder in Chongqing Women’s Compulsory Drug Rehabilitation Center during the rehabilitation consolidation period were surveyed.

In order to ensure the validity and reliability of the questionnaire, all the questionnaires of drug users were sent out by the supervisors of the drug rehabilitation center on their behalf. The questionnaires were filled out and collected with the dormitory as a unit, and all the participants signed the informed consent before filling in. A total of 500 questionnaires were sent out and 487 questionnaires were collected, with a collection rate of 97.4%. Twelve invalid questionnaires were excluded, and 465 questionnaires were finally valid, with an effective rate of 95.4%. The subjects were (35.77 ± 10.29) years old, (1.62 ± 0.64) m in height, (57.7 ± 8.51) kg in weight, (7.76 ± 7.24) years in drug abuse, 214 in traditional drugs, 119 in new drugs and 132 in mixed drugs (see Table 2).

**TABLE 2:** Basic characteristics of Subjects.

**Demographic Variable**		**Mean ± SD/n (%)**	**Drug use-related data**		**Mean ± SD/n (%)**
Age(years)		35.77 ± 10.29	Drug types	Traditional drug	214(46.02%)
Body height(m)		1.62 ± 0.64		New drug	119(25.59%)
Body Weight(kg)		57.70 ± 8.51		Mixed drug	132(28.39%)
Educational status	Primary school or below	107(23.01%)	Mainly drug used	Cannabis	60(12.90%)
	Junior	115(24.73%)		Heroin	95(20.43%)
	Senior	154(33.12%)		Cocaine	42(9.03%)
	College or above	89(19.14%)		Methomphetamine	93(2.00%)
Career	Unemployed	141(30.32%)		K powder	18(3.87%)
	Self-employed	135(29.03%)		Others	157(33.76%)
	Staff	132(28.39%)	Relapse	Once	53(11.40%)
	Manual workers	57(12.26%)		Twice	165(35.48%)
				Three time or more	247(53.12%)
			Drug use years		7.76 ± 7.24
				≤10 years	301(64.7%)
				>10 years	164(35.3%)
			Withdrawal period	≤1 years	163(35.05%)
				1—2 years	203(43.66%)
				>2 years	99(21.29%)
			History of disease	Cardiovascular disease	85(18.28%)
				Physiological disease	46(9.89%)
				Mental disorder	15(3.23%)

This study was conducted according to the guidelines laid down in the Declaration of Helsinki and was approved by the ethics committee of Shanghai University of Sport (102772019RT041). Written informed consent form was obtained from all participants before enrolling them in the study.

### Research Methods

#### Measures

Structured questionnaire design was adopted. On the basis of consulting a large number of research literature, through drafting the preliminary draft, predicting the test, analyzing the results of the preliminary test to complete the final draft of the questionnaire, the main contents include three parts consisting of the physical exercise of drug abusers, the internal inhibition, and the current state of medication cravings.

##### Physical Activity Rating Scale (PARS-3)

Three-question test method revised by Liang (1994) is adopted, namely the intensity, time and frequency of physical activity, 5-point Likert scale is adopted for quantification, scoring from 1 to 5 points, and thus measure the level of participation in physical activity. Physical activity score = activity intensity score × (activity time score – 1) × activity frequency score, score interval 0 to 100 points. The scale of physical activity is: low intensity physical activity ≤19 points, moderate-intensity physical activity ≤20–42 points, and high intensity physical activity ≤43 points. The predictive test of the questionnaire showed that the test–retest reliability was higher, and the correlation coefficient *r* = 0.82.

##### Internal inhibition scale

The internal inhibition scale compiled and revised by Jiang and Lin (2000) was adopted. The scale contains 18 items (e.g., I am very easy to lose my temper, or under the teasing of others, I can do almost anything, etc.), which consists of three dimensions. Using 5-point Likert scale to quantify, the choice of “strongly agree, agree, neither agree nor disagree, disagree, strongly disagree” were scored 1–5 points, the higher the score, the stronger the internal inhibition. The internal consistency test showed that the deliberation Cronbach’s α coefficient was 0.91 (seven items), the self-control Cronbach’s α coefficient was 0.86 (five items), the self-discipline Cronbach’s α coefficient was 0.93 (Six items), and the overall Cronbach’s α coefficient was 0.952. The verification results of measurement model are: χ^2^/df = 1.44, RMSEA = 0.03. AGFI = 0.99, CFI = 0.99, TLI = 0.92, IFI = 0.98, GFI = 0.99. It shows that the questionnaire has good measurement validity and reliability.

##### Drug craving scale

The craving belief scale was compiled by Jiang and Lin (2000). The scale contains 22 items (e.g., I can’t relieve my anxiety without taking drugs, or the desire for drug use is far better than my willpower, etc.), which consists of three dimensions. Using 5-point Likert scale to quantify, the choice of “strongly disagree, disagree, neither disagree nor agree, agree, strongly agree” were scored 1–5 points, the higher the score, the stronger the craving for drugs. Internal consistency test results showed that drug cognition Cronbach’s αcoefficient was 0.95 (10 items), irrational belief Cronbach α coefficient was 0.94 (seven items),craving degree Cronbach’s α coefficient was 0.94 (five items), and overall Cronbach’s α coefficient was 0.963. The validation results of the measurement model are: χ^2^/df = 1.24, RMSEA = 0.03, AGFI = 0.99, CFI = 0.99, TLI = 0.92, IFI = 0.98, GFI = 0.99. It shows that the questionnaire has better measurement validity and reliability.

#### Statistical Analysis

Statistical analysis is carried out to the data with SSPS21.0, including variance analysis, correlation analysis, regression analysis, etc.; AMOS21.0 is adopted to construct the model and analyze the path, and the mediation effect is discussed according to Anderson’s two-step procedure. The significant level of all indicators was set at α = 0.05.

## Results

### Differences Between the Types and Duration of Drug Abuse on Internal Inhibition and Drug Craving of Women With Substance Use Disorder

Based on the characteristics of the sample, this study classified the types of drugs used into three types: traditional drugs, new drugs, and mixed drugs. Traditional drugs refer to narcotic drugs that are extracted from natural plants and have sedative and acesodyne effects, which can make person addicted, mainly including opium, heroin, marijuana, etc.; new drugs refer to artificial chemical synthesis such as hallucinogen, stimulants, etc., which can directly act on the central nervous system, produce excitatory or inhibitory effects, irreversible damage to the brain, mainly including methamphetamine, K powder (mainly ketamine), etc.; mixed drugs refers to the use of the combination of two or more traditional and new drugs for a certain period of time To further explore the effects of years of drug use on internal inhibition and drug craving, the years of drug use were recoded into two categories: (1) 10 years or less (group A); (2) more than 10 years (group B).

**FIGURE 2:**
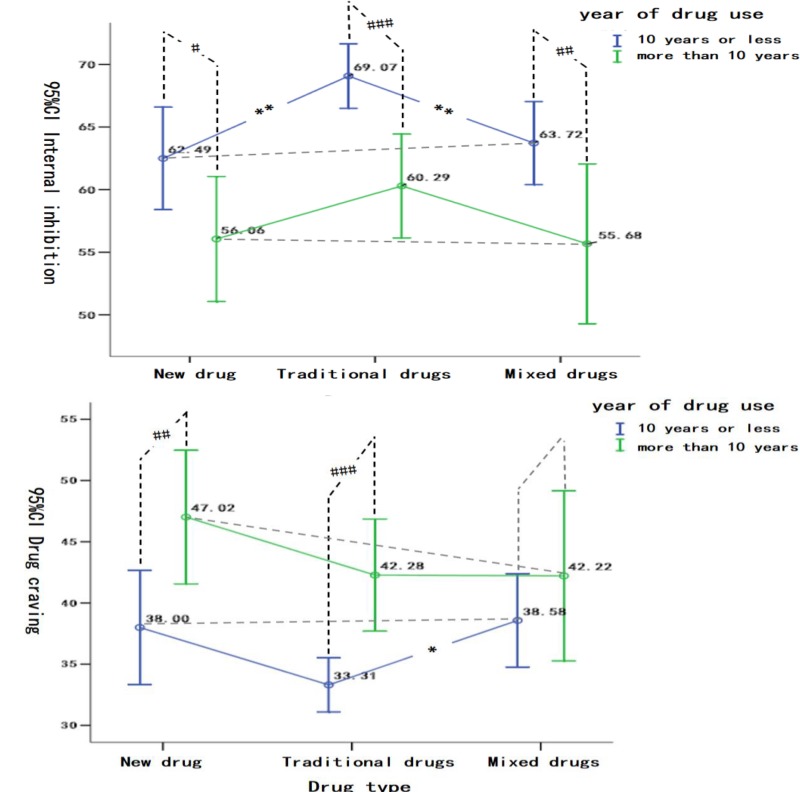
The Differences of the effects of Drug Types and Years in Internal Inhibition and Drug Craving. Within-group comparisons were presented as “#”, i.e., ^#^*P* < 0.05, ^##^*P* < 0.01, ^###^*P* < 0.001; Between-group comparisons were presented as ^∗^, ^∗^*P* < 0.05, ^∗∗^*P* < 0.01, ^∗∗∗^*P* < 0.001.

Figure 2 shows that the different types and drug use years have significant differences in the internal inhibition and drug craving of women with substance use disorder. In terms of internal inhibition, by within-group comparisons, it has been found that: (1) in the new drug users, there were significant differences in the internal inhibition in women with different drug use years (Group A > Group B, *T* = 2.01, *P* < 0.05); (2) in the traditional drug users, the internal inhibition of women with different drug use years was significant difference (Group A > Group B, *T* = 3.75, *P* < 0.001); (3) in the mixed drug users, there were significant differences in the internal inhibition of women with different drug use years (Group A > Group B, *T* = 2.42, *P* < 0.01) by within-group comparisons, it has been found that in the Group A, significant differences occurred among different types of drug users (*F* = 5.15, *P* < 0.01, traditional drug users > new drug users, traditional drug users > mixed drug users), however, there was no significant difference between the new drug users and the mixed drug users (*P* > 0.05), (2) in the Group B, there was no significant difference in the internal inhibition of women with different drug use types (*F* = 1.18, *P* > 0.05).

In terms of drug graving, by within-group comparisons, it has been found that: (1) in new drug users, there were significant differences in drug graving among women with different drug use years (Group A < Group B, *T* = −2.52, *P* < 0.01), and (2) in traditional drug users, there were significant differences in drug graving among women with different drug use years (Group A < Group B, *T* = −3.96, *P* < 0.001), and (3) in mixed drug users, there was no significant difference in drug craving among women with different drug use years (*T* = −0.97, *P* > 0.05). By between-group comparisons, the results showed that: (1) there were significant differences in drug craving between traditional drug users and mixed drug users (*F* = 3.51, *P* < 0.05, mixed drug users > traditional drug users), but there was no significant difference between new drug users and traditional drug users (*P* > 0.05), new drug users and mixed drug users(*P* > 0.05), (2) there was no significant difference in drug craving among women of different types of drug user (*F* = 1.00, *P* > 0.05).

### Differences of Internal Inhibition and Drug Craving of Women With Substance Use Disorder by Physical Activity Intensity

When processing the data, we found that none of the women with substance use disorder reached the high intensity activity, so only two groups of members were obtained, and the score of low-intensity activity group was ≤19 points; moderate-intensity activity group: 20–42 points.

**FIGURE 3:**
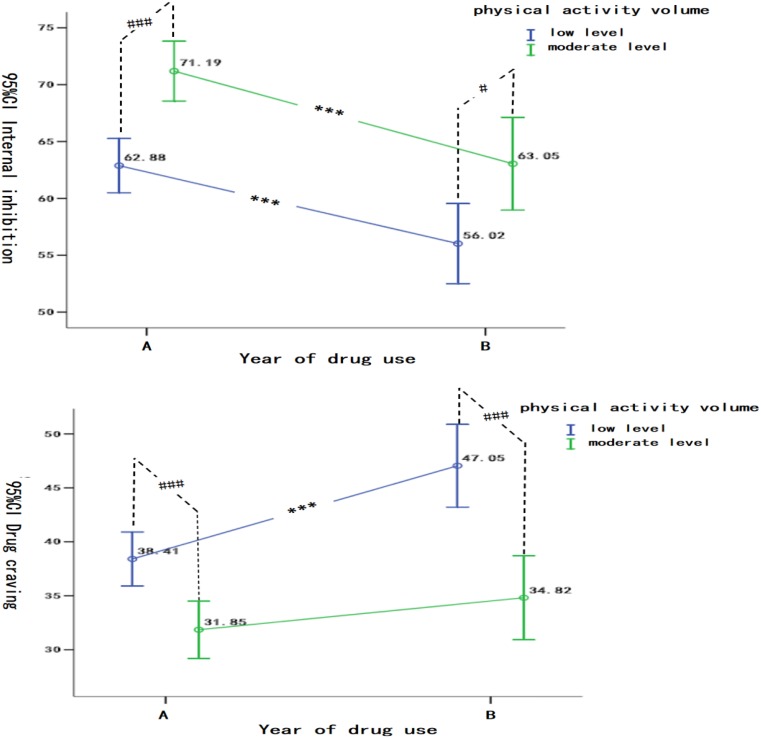
The differences of the Effects of Physical Exercise Volume and Years of drug use in internal inhibition and drug craving. Between-group comparisons were presented as #, i.e., ^#^*P* < 0.05, ^##^*P* < 0.01, ^###^*P* < 0.001; inter-group comparisons were expressed as ^∗^, i.e.,^∗^*P* < 0.05, ^∗∗^*P* < 0.01, ^∗∗∗^*P* < 0.001, and no label for no difference. A: drug use history for 10 years or less, and B: drug use history for more than 10 years.

In terms of internal inhibition, by within-group comparisons, Figure 3 shows that: (1) when the drug use period was 10 years or less, there were significant differences in the internal inhibition of women with different physical exercise volume (moderate level > low level, *T* = −4.42, *P* < 0.001), and (2) when the drug use period was more than 10 years, there was a significant difference in the internal inhibition of women between different exercise volume (moderate exercise level > low exercise level, *T* = −2.61, *P* < 0.05). By between-group comparisons, it has been found that: (1) there were significant differences in the internal inhibition of women with different drug use years in low exercise volume (Group A > Group B, *T* = 3.31, *P* < 0.001), (2) there were significant differences in the internal inhibition of women with different drug use years in moderate exercise volume (Group A < Group B, *T* = 3.32, *P* < 0.001).

In terms of drug craving, by within-group comparisons, it has been showed that: (1) when drug use period was 10 years or less, there were significant differences in drug craving of women with different physical exercise volume (low exercise level > moderate exercise level, *T* = 3.55, *P* < 0.001), and (2) when drug use period was more than 10 years, there were significant differences in drug craving of women with different physical exercise volume (low exercise level > moderate exercise level, *T* = 4.48, *P* < 0.001). By between-group comparisons, it has been found that: (1) there were significant differences in drug craving of women with different drug use years (Group B > Group A, *T* = −3.90, *P* < 0.001), (2) there was no significant difference in drug craving of women with moderate exercise volume (*T* = −1.21, *P* > 0.05).

### Correlation Among Physical Activity Intensity, Internal Inhibition, and Drug Craving

Table 3 shows that the physical activity intensity was negatively correlated with drug craving (*r* = −0.28), the internal inhibition was negatively correlated with drug craving (*r* = −0.42), and the physical activity intensity was positively correlated with the internal inhibition (*r* = 0.31). The results show that there is a significant correlation among the variables, which provides an ideal footstone for the subsequent test of mediating effect. Therefore, the hypothesis X1, X2, and X3 in this study has all been confirmed.

**TABLE 3:** Analysis of the correlation among the physical activity intensity, internal inhibition and drug craving (*N* = 465).

	***M***	***SD***	**1**	**2**	**3**	**4**	**5**	**6**	**7**
(1) Amount of physical activity	20.27	9.56	–						
(2) Internal inhibition	63.09	17.36	0.31^∗∗∗^	–					
(3) Drug craving	38.75	18.25	−0.28^∗∗∗^	−0.42^∗∗∗^	–				
(4) Drug Cognition	16.65	8.92	−0.23^∗∗∗^	−0.35^∗∗∗^	0.92^∗∗∗^	–			
(5) Irrational belief	14.41	7.25	−0.25^∗∗∗^	−0.38^∗∗∗^	0.87^∗∗∗^	0.65^∗∗∗^	–		
(6) Craving degree	7.69	4.50	−0.28^∗∗∗^	−0.40^∗∗∗^	0.83^∗∗∗^	0.69^∗∗∗^	0.63^∗∗∗^	–	
(7) Years of drug abuse	7.76	7.24	−0.07	−0.28^∗∗∗^	0.26^∗∗∗^	0.18^∗∗∗^	0.22^∗∗∗^	0.25^∗∗∗^	–

*^∗^*P* < 0.05, ^∗∗^*P* < 0.01, ^∗∗∗^*P* < 0.001.*

### Test of Mediating Effect of Physical Activity Intensity on Drug Craving

**FIGURE 4:**
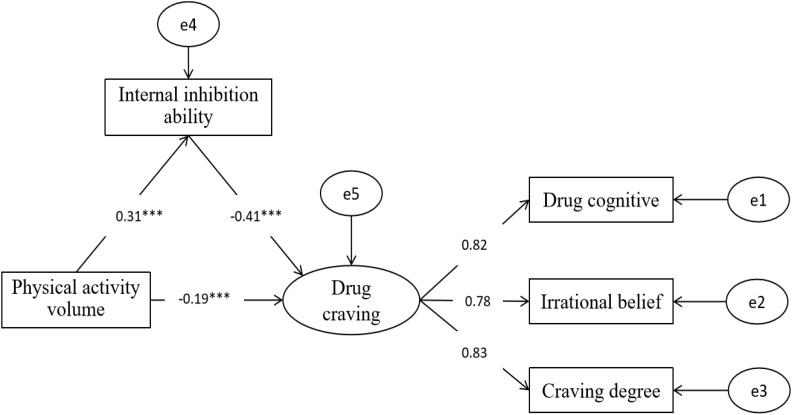
Model diagram of mediating effect of internal inhibition ability on physical activity and drug craving (The digital listed in the figure shows the direct correlation coefficient between variables, it also confirmed the hypothesis in Figure 1).

Figure 4 shows: Structural equation model was used to examine the mediating effect of internal inhibition between physical activity and drug craving. The fitting indexes of structural equation model are as follows:χ^2^/df = 1.12, RMSEA = 0.02, GFI = 0.99, TLI = 0.99, CFI = 0.99, NFI = 0.99, AGFI = 0.98. It indicates that the model fitness is suitable for testing.

Firstly, taking the physical activity intensity as predictive variable and drug craving as dependent variable, the direct effect path coefficient of physical activity intensity on drug craving (β1 = −0.31, *P* < 0.001) is significant. Secondly, after adding the internal inhibition between the physical activity intensity and drug craving as the mediating variable, the path coefficients of physical activity and internal inhibition (β = 0.31, *SE* = 0.08, *P* < 0.001) are significant, and the path coefficient of internal inhibition and drug craving (β = −0.41, *SE* = 0.02, *P* < 0.001) are significant. It is noteworthy that after adding mediating variable, the path coefficient of physical activity volume and drug craving have been decrease from (β1 = −0.31, *P* < 0.001) to (β2 = −0.19, SE = 0.04, *P* < 0.001), and the path coefficient remain reached significance, it indicated the partial medicating effect of internal inhibition between physical activity and drug craving, the medicating effect is (β = −0.13, *P* < 0.001), so the total ratio of the mediating effect is 41%.Therefore, the hypothesis X4 of this study has been confirmed.

The path model shows that physical activity can directly and negatively predict drug craving. Internal inhibition can directly and negatively predict drug craving. The physical activity intensity can directly and positively predict the internal inhibition. The physical activity intensity can also produce indirect effect on drug craving through internal inhibition, that is, the greater the physical activity intensity, the stronger the internal inhibition, and then the drug craving can be effectively reduced.

## Discussion and Analysis

### Analysis of the Influencing Factors of Internal Inhibition and Drug Craving

This study found that the type of drug abuse has a significant impact on internal inhibition and drug craving. Female who uses traditional drugs has higher internal inhibition than those who use new drugs and mixed drugs, which is similar to previous studies (Du and Zhao, 2014). Female who takes new drugs has a higher craving for drugs than those who take traditional drugs. In other words, the use of new drugs has a serious impact on women’s internal inhibition, and irreversible damage to the brain of the drug users, and will lead to increased drug craving (Yang, 2010; Wang T.Y. et al., 2015). Additionally, the duration of drug abuse is another cause for the difference between internal inhibition and drug craving. With the increase of drug addiction years, the degree of brain inhibition function damage increases and the internal inhibition weakens. At the same time, it will increase the craving for drugs, generate the impulse to continue drug abuse, and eventually lead to repeated relapse behavior. Some studies have pointed out that the longer the time of drug abuse, the stronger the drug dependence of drug users (Chen et al., 2017), the stronger the body’s tolerance to drugs (Jiang, 2006), the lower the cognitive level, and that female are significantly lower than male (Zhang et al., 2016).

Similarly, the physical activity intensity is another important reason for the difference in the internal inhibition and drug craving among women with substance use disorder. The results of this study show that the total physical activity intensity of women with substance use disorder in Chongqing is at a medium or low level. In fact, this special group (women with substance use disorder) restricts their own level of physical activity, so that it is difficult to reach a high level of activity. Women in the moderate-intensity activity group had significantly higher internal inhibition than those in the low-intensity activity group, and their craving for drugs was significantly lower than that in the low-intensity activity group, which was consistent with the earlier studies. For example, the inverted U-shaped theory holds that different intensity of activity has different effects on inhibition ability (Yerkes and Dodson, 1908). Compared with low-intensity and high-intensity aerobic activity, acute aerobic activity at moderate intensity has the greatest promoting effect on human inhibition ability, which can effectively improve cognitive processing efficiency, thereby reducing drug craving and corresponding drug-seeking behavior (Wang T.Y. et al., 2015).

### The Direct Influence of Physical Activity on Drug Craving

The correlation analysis showed that the physical activity intensity was negatively correlated with drug craving of women with substance use disorder. Within a certain range, with the increase of physical activity, it will reduce the drug craving of substance users, which is similar to previous studies (Taylor et al., 2007; Kinnunen et al., 2008; Zhao et al., 2018). Of the college students addicted to smoking, the greater the physical activity intensity, the lower the dependence on smoking (Zhu et al., 2014). Of the MA users, the researchers found that the aerobic activity at moderate intensity for 12 weeks was more effective in reducing drug craving and improving the emotional disorders of the substance users than activity intervention for 6 and 9 weeks (Wang and Zhu, 2017). Specifically, 6 weeks of moderate-intensity activity can reduce drug intake during the initial and maintenance stages of drugs. In abstinence stage, 12 weeks of moderate-intensity activity can significantly reduce drug craving (Friedman and Miyake, 2003). Moderate-intensity activity is most beneficial to the dose-response relationship between drug craving and inhibition control in drug users (Wang D.S. et al., 2015), and can significantly reduce drug craving and drug-seeking behavior in drug users (Sinyor et al., 1982). In the field of neurobiology, there is a similar viewpoint that long-term and sustained moderate-intensity endurance activity can keep the dopamine conversion rate rising steadily, and maintain the dose effect of “increase immediately after activity – decrease slowly after 24 h,” thus delaying the relapse interval (Fisher et al., 2004). If the stimulation of physical activity is too large and the time is too long, it will cause metabolic imbalance or metabolic impotence. The synthesis rate of DA far exceeds the metabolic rate, which leads to the “counter-promotion” effect of inducing relapse impulse (Zhao et al., 2018). Therefore, under the condition of controlling the physical activity intensity, the purpose of reducing drug craving can be achieved by increasing the physical activity intensity of drug users, and this study confirms the viewpoint of the above research.

### Mediating Effect of Internal Inhibition on Physical Activity and Drug Craving

Path model shows that physical activity is positively correlated with internal inhibition, while internal inhibition is negatively correlated with drug craving, which is consistent with previous research results (Baumeister et al., 1996; Muraven, 2010; Zhu et al., 2014). With the increase of the physical activity intensity, the internal inhibition of drug-dependent patients will be effectively enhanced, which indicates that physical activity and internal inhibition are mutually reinforcing and influencing, which has also reached a consistent conclusion in the normal population (Roessler, 2010; Barenberg et al., 2011; Drollette et al., 2012). Of course, this does not mean that the higher the physical activity intensity, the better it will be. Especially for drug users, if the physical activity intensity is too large, the relapse impulse will not be effectively suppressed, and even have the opposite side effects (Zhao et al., 2018). Therefore, some studies have pointed out that compared with low-intensity activity, moderate-intensity activity can improve the response speed of inhibition task more effectively (Joyce et al., 2009), and acute aerobic activity at moderate intensity can induce the best level of arousal, which has the greatest benefit on cognitive ability (Mcmorris and Hale, 2012). This confirms the findings of this study: between low and moderate-intensity activity, the positive linear relationship between physical activity and internal inhibition is reasonable and scientific, and it also supports and enriches the internal mechanism of inverted U-shaped theory.

In addition, internal inhibition can negatively predict drug craving. Studies have shown that internal inhibition is closely related to drug craving and relapse behavior (Jiang and Lin, 2000; Yang et al., 2007). This has been verified in the study of AIDS drug abusers. There is a significant negative correlation between the internal inhibition of AIDS drug abusers and drug craving (Liu, 2008). That is, the higher the internal inhibition, the lower the drug craving, and the internal inhibition can reduce the occurrence of deviation or criminal behavior. According to the theory of self-control, people with strong self-control ability seldom have bad behaviors, such as smoking, excessive drinking, drug abuse, etc. Through 2 weeks of self-control training, smoking addicts can effectively prolong the time to quit smoking (Roessler, 2010). Similarly, some bad behavior can have a negative effect on inhibition function. For example, long-term drug abuse can lead to disorders in the prefrontal area of the brain. Even after withdrawal, the brain function of drug users will be impaired, inhibition and other advanced cognitive functions will still be abnormal (Yuan et al., 2010; Peterson et al., 2014). Therefore, this study establishes a path mechanism between physical activity intensity, internal inhibition and drug craving.

In this study, internal inhibition was used as a mediating variable to explore the relationship between physical activity and drug craving. The results showed that internal inhibition played a part of the mediating role. This shows that physical activity can not only directly reduce drug users’ drug craving, but also achieve the rehabilitation benefits of physical detoxification through the mediating effect of internal inhibition. Studies have shown that physical activity can inhibit drug craving by activating the anterior cingulate gyrus and improving cognitive function (Zhao et al., 2017). Although some research theories suggest that high intensity activity can maximize the inhibition ability, and low-intensity acute aerobic activity has a higher accuracy in the inhibition task than moderate-intensity and high intensity (Allard et al., 1989). However, it should be noted that it is very difficult for drug users to achieve high intensity activity, especially among female addicts, which has been fully proved in the practical investigation of this study. For drug users, moderate physical activity intensity plays a significant role in drug craving and drug seeking behavior (Sinyor et al., 1982). When the physical activity intensity is controlled in the moderate-intensity range, with the increase of the physical activity intensity, the internal inhibition will be strengthened, so the resistance to drug and other substances will be stronger, and the more conducive to reducing drug craving. This path mechanism has been supported by many empirical studies in the above studies (Liang, 1994; Peterson and Johnstone, 1995; Yang et al., 2007; Mcmorris and Hale, 2012; Wang and Zhu, 2017). Therefore, through combing the previous studies, this study believes that in the mechanism of activity drug treatment, physical activity should be carried out according to the actual situation, and meanwhile strengthen the monitoring and training of drug users’ internal inhibition, and formulate the optimal activity intervention scheme, so as to minimize their drug craving and help them return to their families and society as soon as possible.

## Conclusion

(1) Women who take traditional drugs have the strongest internal inhibition, and those who take new drugs have the highest drug craving; the longer the duration of drug abuse, the lower the internal inhibition and the higher the drug craving; women with moderate activity intensity had the strongest internal inhibition and the lowest drug craving.
(2) The physical activity intensity is negatively correlated with drug craving and positively correlated with the internal inhibition, and the internal inhibition is negatively correlated with drug craving.
(3) Internal inhibition plays a partial mediating effect between physical activity intensity and drug craving.

### Limitations and Further Research Directions

This study explores the mediating effect of internal inhibition in the influence of physical activity on women with substance use disorder’ drug craving path for the first time. It shows that physical activity can directly or indirectly reduce drug abuser’s drug craving in the treatment of drug problem in the whole society. To some extent, it reveals the potential path mechanism of physical detoxification. In the follow-up study, we can further explore the following issues:

(1) Since the cross-sectional study is adopted, we get the correlation among variables, but we cannot get a deeper causal relationship. Longitudinal study can be added to the future study to better reveal the causal relationship among variables.
(2) This study focuses on the mediating variables between internal inhibition and physical activity and drug craving, and more mediating or regulating variables can be explored in the future.
(3) The main research object of this study is the influence of physical activity intensity on drug craving of women with substance use disorder, and male drug users can be added as a comparison in the follow-up study.

## Data Availability

All datasets generated for this study are included in the manuscript and/or supplementary files.

## Ethics Statement

Human subject research: the studies involving human participants were reviewed and approved by ethics committee of Shanghai University of Sport (102772019RT041). The patients/participants provided their written informed consent to participate in this study.

## Author Contributions

All authors designed this study. KW and JL carried out the protocol and questionnaire survey. TZ recruited the individuals with drug addicts. YO and JL undertook the statistical analysis and graphical representation of the data. CZ and YL revised the draft. All authors contributed to and approved the final manuscript.

## Conflict of Interest Statement

The authors declare that the research was conducted in the absence of any commercial or financial relationships that could be construed as a potential conflict of interest.
